# Mononuclear cells modulate the activity of pancreatic stellate cells which in turn promote fibrosis and inflammation in chronic pancreatitis

**DOI:** 10.1186/1479-5876-5-63

**Published:** 2007-12-05

**Authors:** Christoph W Michalski, Andre Gorbachevski, Mert Erkan, Carolin Reiser, Stefanie Deucker, Frank Bergmann, Thomas Giese, Markus Weigand, Nathalia A Giese, Helmut Friess, Jörg Kleeff

**Affiliations:** 1 Department of Surgery, Technische Universität München, Munich, Germany; 2 Department of General Surgery, University of Heidelberg, Germany; 3 Institute of Pathology, University of Heidelberg, Germany; 4 Institute of Immunology, University of Heidelberg, Germany; 5 Department of Anaesthesiology, University of Heidelberg, Germany

## Abstract

**Background:**

Interactions between mononuclear cells and activated pancreatic myofibroblasts (pancreatic stellate cells; PSC) may contribute to inflammation and fibrosis in chronic pancreatitis (CP).

**Methods:**

Markers of fibrosis and inflammation were concomitantly analysed by immunohistochemistry in chronic pancreatitis tissues. In vitro, PSC were stimulated with TNFalpha and LPS. Primary human blood mononuclear cells (PBMC) and PSC were cocultured, followed by analysis of cytokines and extracellular matrix (ECM) proteins. PBMC were derived from healthy donors and CP and septic shock patients.

**Results:**

In areas of mononuclear cell infiltration in chronic pancreatitis tissues, there was decreased immunoreactivity for collagen1 and fibronectin, in contrast to areas with sparse mononuclear cells, although PSC were detectable in both areas. LPS and TNFalpha induced collagen1 and fibronectin levels as well as the matrix degradation enzyme MMP-1. Coculture experiments with PSC and PBMC revealed increased fibronectin secretion induced by PBMC. In addition, donor and CP PBMC significantly induced an increase in IL-6, MCP-1 and TGFbeta levels under coculture conditions. Determination of the source of cytokines and ECM proteins by mRNA expression analysis confirmed PSC as major contributors of ECM production. The increase in cytokine expression was PBMC- and also PSC-derived.

**Conclusion:**

Mononuclear cells modulate the activity of pancreatic stellate cells, which may in turn promote fibrosis and inflammation.

## Background

The exact pathogenesis of chronic pancreatitis (CP) is unknown, but the disease results from an injury to a pancreatic cell population, which then activates pancreatic stellate cells (PSC) as a final common response. This process is associated with excessive accumulation of extracellular matrix (ECM) proteins by PSC and a massive infiltration of mononuclear cells [1-6]. Clinically, CP is accompanied by a severe pain syndrome and by a loss of pancreatic function (both exocrine and endocrine). PSC have been determined as the major source of ECM protein production, and have also been shown to produce cytokines and chemokines [7-11]. Thus, anti-fibrogenic therapies aim to reduce the activity of PSC in order to inhibit the accumulation of ECM proteins and also to prevent digestion of the basement membranes, which allows PSC to enter inflammatory areas and to excessively deposit ECM.

In liver cirrhosis, tissue macrophages derived from blood mononuclear cells have been shown to influence fibrogenesis by activating hepatic myofibroblasts [12], while suppression of macrophage infiltration inhibits hepatic stellate cell activation and thus liver fibrogenesis. During fibrogenesis, local inflammation may not be initiated by apoptosis/necrosis of parenchymal cells but by resident and recruited inflammatory cells; these inflammatory cells, once in an active state, release cytokines which in turn activate stellate cells [13,14].

Recent studies have investigated the response of hepatic stellate cells to lymphocyte subsets, which can be divided into Th1 and Th2 predominant [15]. Different Th1 or Th2 subsets seem to modulate the fibrotic response towards anti- or pro-fibrogenesis, thus resulting in an apparent IL-10 paradox: IL-10 is anti-fibrogenic but is produced during a pro-fibrotic Th2 immune response [16]. Mononuclear cells within fibrotic areas have been shown to secrete a wide variety of agents which induce matrix generation/degradation [17-19], but there are no data on different responses of PSC to PBMC from various sources – i.e., healthy donors and CP patients.

In CP, a generalized immune response has been suggested due to increased expression of TNFalpha and its receptor on PBMC and which was associated with PSC cytotoxicity [20]. Furthermore, it has been shown that LPS-activated macrophages stimulate the synthesis of ECM proteins by PSC [21]. In rats, suppression of macrophage infiltration inhibited activation of hepatic stellate cells [12]. Similarly, it has been shown that both pro- and anti-inflammatory cytokines, such as PDGF and TGFbeta, activate PSC and thus contribute to pancreatic fibrogenesis [22]. TGFbeta was subsequently confirmed as a key regulator of ECM production and PSC proliferation due to its inhibition of MMPs in an autocrine fashion which enhanced fibrogenesis by reducing collagen degradation [23].

In terms of transcriptional regulation, nuclear factor kappaB (NFkB) is a well-characterized transcription factor which is activated by oxidative stress or by TNFalpha, and which activates key anti-fibrotic genes such as MMP-2. Interestingly, inhibition of NFkB by IkB has been shown to decrease IL-6 and ICAM-1 levels in rat hepatic stellate cells during experimental liver injury [24]. Thus in many ways, lymphocytes modulate fibrogenesis depending on their activation state, either towards anti- or pro-fibrosis. Clinically, it is hypothesized that immune suppression accelerates the development and progression of fibrosis, underlining the importance of immune regulation for stellate cell function [25-27].

Understanding the pathobiological mechanisms underlying activation states of immune cells and PSC, as well as their interactions which are associated with the development of chronic pancreatitis, is therefore a prerequisite for the design of novel therapeutic approaches. Here, we demonstrate the significance of an interaction between PBMC and PSC which influences fibrogenesis and inflammation in chronic pancreatitis.

## Patients, Materials and Methods

### Patients and tissue sampling

Patients with chronic pancreatitis (n = 13), patients with sepsis (n = 4), and age- and sex-matched healthy adults (n = 10) were eligible for enrollment in the study. Blood samples were collected preoperatively from CP patients or on the intensive care unit from septic shock patients. Tissue samples were collected during pancreatic resections for CP. These were either immediately processed for isolation of primary human pancreatic stellate cells or snap frozen at -80°C or formalin fixed and paraffin embedded. The use of human material for analysis was approved by the local ethics committee (University of Heidelberg, Germany), and written informed consent was obtained prior to the operation and the blood sampling.

### Immunohistochemistry

Immunohistochemistry was performed as described previously [28,29]. Briefly, consecutive sections from chronic pancreatitis patients (n = 10) were stained for collagen1, fibronectin, alpha-SMA, CD31, CD45 and vimentin. Primary antibodies against collagen1 (diluted 1:600; ab6308, Abcam, Cambridge, UK), fibronectin (diluted 1:500; F3648, Sigma Aldrich, Taufkirchen, Germany), alpha-SMA (diluted 1:500; M0851, DAKO Cytomation, Hamburg, Germany), CD-31 (diluted 1:50; M0823, DAKO Cytomation), CD45 (ready to use; N1514, clone 2B11&PD7/26, DAKO Cytomation) and vimentin (diluted 1:500; M7020, DAKO Cytomation) were applied at 4°C overnight. Secondary antibodies (anti-mouse) for collagen1, alpha-SMA, CD31, CD45, and vimentin and (anti-rabbit) for fibronectin were purchased from DAKO Cytomation as EnVision+ ready-to-use solutions.

### Human primary PSC and PBMC isolation and culture

Human PSC isolation and culture were performed as described previously [8,28]. PBMC were isolated from heparinized blood by ficoll density gradient separation (Histopaque^®^-1077, Sigma-Aldrich, Taufkirchen, Germany) as previously described [30].

### Treatment of PSC with TNF-alpha and LPS

Sister clones of PSC were treated with TGFbeta1 (5 ng/ml, GF111 Chemicon Temecula, CA, USA), TNFalpha (1 ng/ml, GF023 Chemicon Temecula, CA, USA), and LPS (1 ng/ml). Collection of RNA was performed at 6 and 24 hours as described before [31].

### Coculture of PSC and PBMC

Primary human PSC were cocultured with freshly isolated PBMC using cell culture inserts with 1.0 μm pore size according to the manufacturer's instructions (BD Falcon, city, NJ, USA). PSC were plated on the bottom of 6-well cell culture plates at a density of 25,000/cm^2 ^in 3 ml of DMEM/Ham's F12 in the presence of 10% FCS 24 hours prior to the onset of coculture. Thereafter, medium was changed to DMEM/Ham's F12 with 0.5% FCS and freshly isolated human PBMCs were seeded on the membrane insert in 2 ml of the same culture medium. The ratio of PBMC/PSC was 15:1. After overnight incubation (24 hours), the supernatants from PSC and PBMC were mixed and immediately frozen at -80°C for ELISA experiments; the cells were processed and frozen at -80°C prior to analysis by QRT-PCR and immunoblotting.

### Quantitative real-time RT-PCR

All reagents and equipment for mRNA/cDNA preparation were purchased from Roche Applied Science Diagnostics (Mannheim, Germany). QRT-PCR was performed as described previously [31]. All primers were obtained from Search-LC (Heidelberg, Germany).

### Immunoblot analysis

For immunoblot analyses of cocultured PSC and PBMC as well as LPS- or TNFalpha-treated PSC, cells were processed as described [28,29]. Primary antibodies against collagen type-1 (diluted 1:2000; sc-28657, Santa Cruz Biotechnology, Santa Cruz, CA, USA), fibronectin (1:10,000; F3648, Sigma Aldrich, Taufkirchen, Germany), alphaSMA (1:10,000; M0851, DAKO Cytomation, Hamburg, Germany) and gamma-tubulin as an equal loading control (1:5,000; sc-7396, Santa Cruz Biotechnology) were applied overnight at 4°C; secondary antibodies (collagen type-1 diluted 1:2,000; fibronectin, alphaSMA and gamma-tubulin diluted 1:5,000) were added for one hour at room temperature. Densitometry was performed as described previously [28].

### Enzyme-Linked Immunosorbent Assay (ELISA)

To determine levels of MCP-1, IL-6 and TGFbeta in coculture supernatants, commercial ELISA kits were used according to the manufacturer's instructions (OptEIA™ ELISA kits for human MCP-1 and IL-6 from BD Biosciences [BD, Heidelberg, Germany]; ELISA DuoSet^® ^human TGFbeta kit from R&D Systems [R&D, Abingdon, UK]).

### Nuclear extracts and NFkB p65 ELISA

To isolate the nuclear protein fraction and to measure nuclear NFkB p65 levels, cells were processed after 2 hours of coculture using a commercial TransAM™ NF-kB family ELISA-based kit according to the manufacturer's instructions (Active Motif, Rixensart, Belgium).

### Statistical analysis

Statistical analysis was performed using GraphPad Prism 4 Software (GraphPad, San Diego, CA, USA). A paired t-test was used for analysis of densitometries. The Mann-Whitney U test was used for comparisons of cytokine levels in cell culture supernatants and for comparisons of cytokine expression in PBMC. Analysis of variance (Kruskal-Wallis test) was performed for comparisons of differences of PSC mRNA expression levels, followed by a post-hoc Dunn's multiple comparison test. The level of statistical significance was set at p < 0.05.

## Results

### Suppression of ECM production in mononuclear cell infiltrates in chronic pancreatitis

**Figure 1:**
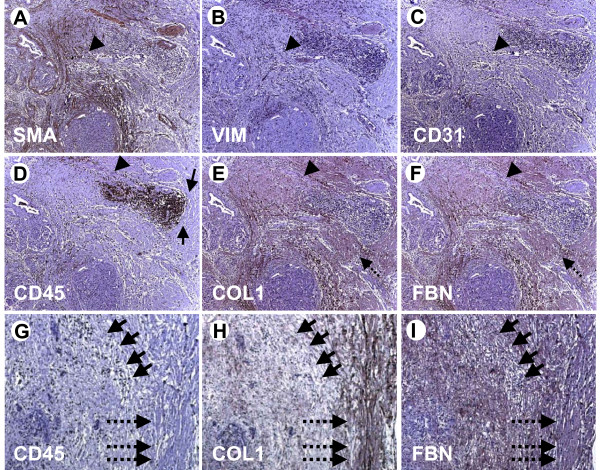
**Immunohistochemistry of human chronic pancreatitis tissue specimens**. Consecutive sections of chronic pancreatitis tissues were subjected to immunohistochemistry using antibodies against alphaSMA (A; SMA), vimentin (B; VIM), CD31 (C), CD45 (D), collagen1 (E; COL) and fibronectin (F; FBN). Arrows: area around packed clusters of mononuclear cells. Arrowheads in A-C: pancreatic stellate cells (SMA- and VIM-positive, CD31-negative). Arrowhead in D: loosely distributed area of mononuclear cell infiltration. Arrowhead in E&F: areas of less intense immunoreactivities for collagen1 and fibronectin. Dotted arrows: Areas of strong collagen1 and fibronectin staining. Original magnification: ×50. (G) Arrows: areas of loosely distributed/single mononuclear cells, dotted arrows: area without mononuclear cells. (H&I) Arrows/dotted arrows: corresponding areas to (G).

**Figure 2:**
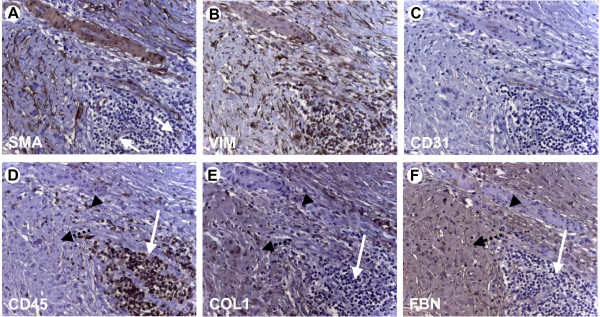
**Details of immunohistochemical analysis of chronic pancreatitis tissues**. (A&B) Pancreatic stellate cells are seen within clusters of mononuclear cell infiltration. (A) Arrows: stellate cells. (C) To exclude that alphaSMA- (SMA) and vimentin-positive (VIM) structures are endothelia, CD31 staining was performed. (D-F) In areas of loosely packed mononuclear cells (arrowhead in D) and packed mononuclear cells (white arrow in D), there is sparse immunoreactivity for collagen1 (arrowhead and white arrow in E) and moderate staining for fibronectin (arrowhead and white arrow in F). In areas with a low number of CD45-positive mononuclear cells (dotted arrow in D), the staining intensities for collagen1 (dotted arrow in E; COL) and fibronectin (dotted arrow in F; FBN) are stronger. Original magnification: ×200.

To judge the pattern of extracellular matrix protein distribution within and in the vicinity of mononuclear cell infiltrates in human chronic pancreatitis tissues (n = 10), immunohistochemistry was performed with antibodies against mononuclear cells (CD45; MNC), activated pancreatic stellate cells (alpha smooth muscle actin, alpha-SMA; vimentin; activated PSC), and the ECM proteins fibronectin and collagen1. To distinguish between PSC and small vessels, the endothelial marker CD31 was used. We then analysed patterns of stellate cells, mononuclear cells and matrix deposition. Pancreatic stellate cell activity (alpha-SMA-positive and vimentin-positive cells: Figure 1A &1B; for endothelial staining see Figure 1C) and matrix deposition were different around different patterns of mononuclear cell infiltration, namely, around and within 1) clusters (Figure 1D; arrows), 2) loosely distributed mononuclear cells (Figure 1D; arrowhead) and 3) single mononuclear cells (Figure 1A–C; arrowheads; PSC: alphaSMA-positive and vimentin-positive, but CD31-negative). Activated PSC deposit extracellular matrix (ECM; Figure 1D, arrows; Figure 1E &1F, dotted arrows) and as expected, PSC are found mainly in areas of inflammation and ECM deposition (Figure 1A &1B; for endothelial staining see Figure 1C). Activated PSC were also found within clusters of mononuclear cells (Figure 2A &2D, arrows; Figure 2B &2C, corresponding vimentin and CD31 stainings) and, therefore, one might expect deposition of ECM within these clusters but none is seen (Figure 2E &2F, arrows) suggesting mononuclear cell suppression. These findings are corroborated by further immunohistochemistries showing that in areas of loosely distributed/single mononuclear cells (Figure 1G, arrows), there is only faint collagen1 and fibronectin staining (Figure 1H &1I, arrows). However, in areas without mononuclear cells (Figure 1G, dotted arrows), there is strong ECM protein staining (Figure 1H &1I, dotted arrows).

### LPS and TNFalpha modulate PSC activity

**Figure 3:**
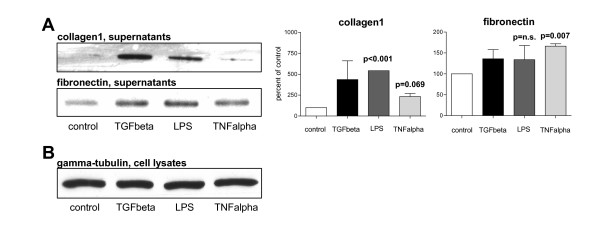
**Immunoblot of PSC cell culture supernatants and cell lysates**. Cell culture supernatants and cell lysates of vehicle-, TGFbeta-, LPS- and TNFalpha-treated PSC were subjected to SDS-PAGE followed by incubation with (A; supernatants) anti-collagen1 and anti-fibronectin antibodies or (B; cell lysates) an anti-gamma-tubulin antibody Densitometry is shown in percent of vehicle-treated control PSC supernatants; n.s. = statistically not significant.

The effects of pro-inflammatory substances on the activation state of PSC were examined by incubation with TNFalpha and lipopolysaccharide (LPS). As shown before [28], analysis of "functional" ECM proteins can be performed on cell culture supernatants. This revealed that TNFalpha and LPS exerted similar effects on PSC. Immunoblot analysis of cell culture supernatants was used to analyze the secretion of the extracellular matrix proteins collagen1 and fibronectin by PSC. These experiments showed that both LPS and TNFalpha increased the secretion of collagen1 and fibronectin (Figure 3A). Furthermore, the matrix degradation enzyme MMP-1 was induced (LPS by 46%; TNFalpha by 454%), while its inhibitor TIMP-2 was suppressed (by 20% and 18%, respectively; data not shown). Densitometry adjusted to control PSC supernatants revealed that LPS increased collagen secretion (Figure 3A, p < 0.001) and induced a trend towards increased fibronectin levels (Figure 3A). TNFalpha induced an increase in collagen1 (p = 0.069) and an increase in fibronectin levels (p = 0.007).

### In vitro increase of ECM production is induced by PBMC

**Figure 4:**
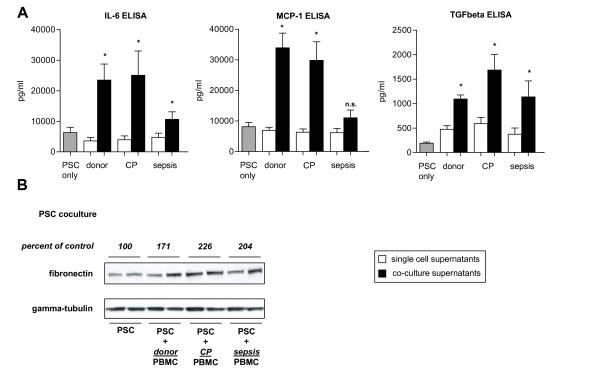
**Analysis of PSC supernatants and cell lysates following coculture with PBMC**. (A) PSC cell culture supernatants were subjected to ELISA following coculture with donor, CP and sepsis PBMC. Hatched bars: PSC supernatants. White bars: Pooled donor, CP or sepsis PBMC and corresponding PSC supernatants. Black bars: coculture supernatants. *p < 0.05 as assessed using the Mann-Whitney U test. (B) Immunoblot analysis of fibronectin in PSC cell lysates. Densitometry shown as percent of control adjusted to the corresponding gamma-tubulin density.

To evaluate whether mononuclear cells alter ECM secretion of cultured primary human PSC in vitro, a co-culture system with PBMC in the upper chamber and PSC in the lower chamber was used. Cytotoxicity and immunological incompatibilities were excluded due to spatial distance between the cells in the co-culture chambers. Here, PBMC derived from healthy donors led to a 71% increase in PSC fibronectin levels after 24 hours of co-incubation (Figure 4B). Using CP PBMC, this effect was more pronounced, with a 126% increase in fibronectin, whereas in septic shock PBMC there was a 104% increase (Figure 4B). To assess whether these effects were dependent on TGFbeta, its levels in cell culture supernatants were analyzed by ELISA. These experiments revealed significantly increased TGFbeta levels (by 130%, 185% and 204%, respectively) following coculture with donor, CP and sepsis PBMC (Figure 4A; p < 0.0001, p = 0.0002 and p = 0.0004, respectively).

### Coculture of PSC and PBMC induces cytokine secretion

To determine the potential of PSC and PBMC in coculture to produce cytokines and chemokines, IL-6 and MCP-1 levels were determined in cell culture supernatants (Figure 4A). Following coculture with donor, CP and sepsis PBMC, a significant increase of IL-6 levels in cell culture supernatants was observed (by 553%, 531% and 124%, with p < 0.0001, p < 0.0001 and p = 0.007, respectively). A similar effect was seen for MCP-1, with significant induction following coculture with donor (390% increase) and CP (370% increase) PBMC (Figure 4A; both p < 0.0001). However, coculture of PBMC derived from sepsis patients with PSC did not alter IL-6 levels (Figure 4A; p = 0.1).

### Both PBMC and PSC contribute to increased cytokine levels

**Figure 5:**
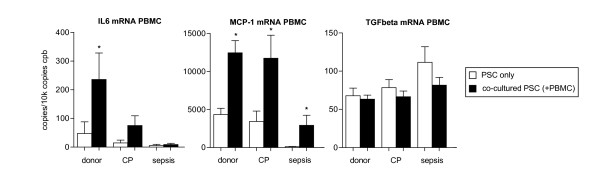
**Messenger-RNA expression levels in single-cell and cocultured PBMC**. PSC alone (without PSC; white bars) and cocultured (with PSC; black bars) PBMC were analyzed by quantitative RT-PCR using primers for IL-6, MCP-1 and TGFbeta. Results are expressed as copies per 10 k copies of cyclophilin B (cpb). *p < 0.05 as assessed using the Mann-Whitney U test.

**Figure 6:**
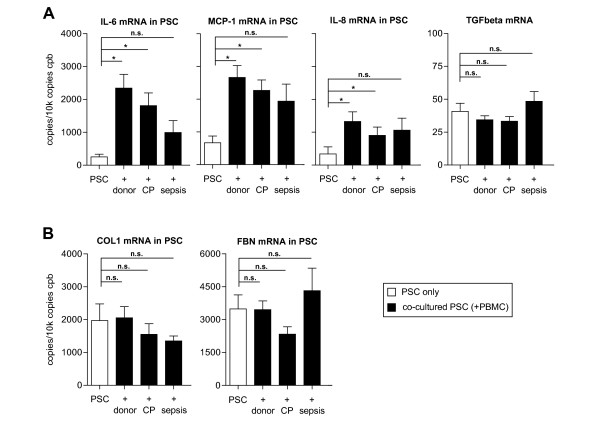
**Messenger-RNA expression levels in single-cell and cocultured PSC**. PSC alone (without PBMC; white bars) and cocultured (with PBMC; black bars) PSC were analyzed by quantitative RT-PCR using primers for IL-6, MCP-1, IL-8 and TGFbeta (A) as well as for collagen1 and fibronectin (B). Results are expressed as copies per 10 k copies of cyclophilin B (cpb). *p < 0.05 as assessed using the Kruskal-Wallis test followed by Dunn's post-hoc test to compare single-cell PSC with cocultured PSC.

Cytokine and chemokine mRNA expression levels were subsequently evaluated in PBMC and PSC to determine the source of increased cytokine/chemokine levels in cell culture supernatants. Altogether, there was an increase in cytokine mRNA expression which was more pronounced in PSC than in PBMC (Figures 5 &6). Analysis of PBMC mRNA expression confirmed the results of the ELISA readouts. Coculture increased IL-6 expression in donor PBMC by 400% (Figure 5; p = 0.005), whereas for CP PBMC there was only a trend towards increased IL-6 levels following coculture (Figure 5; p = 0.186). In contrast to ELISA results, sepsis PBMC did not increase IL-6 at the mRNA level. MCP-1 expression was induced by PBMC coculture with donor, CP and sepsis PBMC (Figure 5; p = 0.0003, p = 0.006 and p = 0.016, respectively). Following coculture with donor and CP PBMC, expression levels of IL-6, MCP-1 and IL-8 were increased in PSC (Figure 6A; all p < 0.05). However, sepsis PBMC did not induce cytokine mRNA expression significantly, although a trend toward an increase was seen (Figure 5A; all p > 0.05).

### Increased ECM protein secretion is not accompanied by increased transcription

A potential influence of late changes in mRNA transcription (24 hours) on the increase of ECM proteins in coculture and the contributing source of these proteins (PSC and/or PBMC) was examined using quantitative RT-PCR. Low-level expression of collagen1 and fibronectin was unaltered in PBMC following coculture (data not shown). TGFbeta expression in PBMC was also unchanged after coculture (Figure 5; p = 0.988, p = 0.3 and p = 0.214, respectively). Analysis of collagen1 and fibronectin mRNA in control and cocultured PSC revealed no changes in expression levels (Figure 6B). Furthermore, TGFbeta expression also remained unchanged after coculture (Figure 6A). Thus, the effect of coculture on ECM protein production seems to be rather independent of the transcription of the respective mRNA moieties, but may involve changes in protein processing/sorting or protein secretion.

### NFkappaB is not involved in signal transduction induced by coculture

NFkappaB levels in the nuclear fraction of PSC and PBMC were determined after two hours of coculture to evaluate potential rapid effects on the transcriptional level. However, there was no difference in NFkappaB transactivation when comparing PBMC and PSC alone with cells in coculture (data not shown).

## Discussion

An important finding of this study is that PBMC-derived substances alter the phenotype of PSC towards a more pro-fibrogenic and pro-inflammatory cell-like phenotype and away from the typical myofibroblast phenotype. This assumption is underlined by increased ECM protein secretion in TNFalpha- and LPS-treated PSC and a concomitant increase in collagen1 and fibronectin as well as in pro-inflammatory cytokines IL-6 and MCP-1 following coculture of PSC with PBMC.

Progression of fibrosis in chronic pancreatitis is accompanied by inflammation and a severe pain syndrome which represents a major clinical challenge [1,9,32,33]. Recently, stellate cells themselves have emerged as inflammatory effectors [34,35]. Upon activation, they release a wide variety of chemokines and cytokines and upregulate the expression of key inflammatory markers [32,36,37]. Since mononuclear cell infiltration is a feature shared by a variety of fibrotic diseases, increasing attention is being paid to investigating the role of immune cells in regulation of stellate cell activity [38,39]. Potential activators of PSC include factors such as cytokines and growth factors [40]. Activated PSC, in turn, produce autocrine factors such as periostin and TGF-β which perpetuate their activation [28].

In our study, immunohistochemical analyses showed an influence of mononuclear cell infiltrates on PSC: There was a strong deposition of ECM proteins around the clusters of immune cells and in areas where immune cells were scarcely distributed in the stroma. On the contrary, within the packed immune clusters, there was weak (fibrononectin) or almost absent (collagen 1, alphaSMA) immunopositivity. These observations might contradict our in vitro data but could also be explained by increased matrix "turnover" activity and a change in PSC phenotype. Moreover, this site specific variance in ECM deposition can be due to the following reasons: 1) The packed immune clusters are actually forming in expanded perivascular spaces as hinted by the presence of central CD31 positive endothelial cells; 2) In vitro, the matrix degradation enzyme MMP-1 was increased together with an increase in ECM proteins collagen1 and fibronectin; 3) High amounts of TNFalpha exert cytotoxic effects on PSC [20] which is probably represented in vivo as a function of their density. Using immunohistochemistry, different phases of immune reaction in the pancreas were seen: in scarred areas where ECM has replaced parenchymal cells, there was also a decline in alphaSMA-positive cells whereas a large number of activated PSC were seen around injured acini and remaining ducts. We have previously shown that periostin, which sustains stellate cell activity, is mostly secreted by PSC around degenerating acini/tubular complexes [28], possibly marking the invasive front of the activated stroma where newly deposited ECM gradually replaces the functional parenchyma. These results are compatible with recent reports which observed that at the end of tissue repair, the reconstructed ECM takes over the mechanical load and myofibroblasts disappear by induction of apoptosis [41]. Our current results underline the change in PSC phenotype towards a more fibroblast-like cell type with immunologic function.

Recently, it has also been shown for hepatic stellate cells that natural killer cells and CD4 T cells may induce anti-fibrosis, whereas CD8 cells are rather pro-fibrotic [42]. Furthermore, cell-to-cell contact of stellate and immune cells seems ultimately not to be necessary to induce such effects, since the results of our coculture set-up point out the importance of secreted mediators. However, our findings may also be due to several other factors: first, we used PBMC, which have only been in direct contact with the inflamed pancreas to a very minor extent; second, upregulation of PSC-produced ECM proteins seems to be a function of PBMC in general, not particularly of PBMC which are derived from CP patients; and third, our study addresses interactions of PBMC and PSC in an artificial set-up which may reflect the experimental processing of both PSC and PBMC. Particularly, while there was a tendency towards an increase in fibronectin production following PSC co-culture with CP and septic shock PBMC, this increase could also have been due to activation of PSC under culture conditions (plastic cell culture dishes which lead to activation of PSC).

In terms of cytokine upregulation by PBMC, coculture seems to be a valid model: it shows that the capacity of PSC to secrete cytokines may be further increased and may thus contribute to the ongoing inflammation in chronic pancreatitis. Interestingly, PBMC derived from septic shock patients lost the ability to induce PSC cytokine expression and secretion which may be due to several reasons. It has been reported that there is a general paralysis of the immune system in sepsis [43-45]. It also seems possible that – due to the strong acute inflammation in sepsis patients – the PBMC activity state has been suppressed. Thus, the secreted mediators which induce PSC activity may be suppressed in this state. There is also an inverse regulation of IL-6 and MCP-1 mRNA induction by PBMC derived from different sources: while the increase in IL-6 expression on the mRNA level was only seen in PBMC from healthy donors, MCP-1 was significantly induced in cocultured PBMC from donors, CP and septic shock patients. The levels of the respective proteins in the supernatants did not reflect the mRNA regulation, which may be due to accumulation in the coculture set-up and the fact that the levels in the supernatants are a result of contributions by both PBMC and PSC.

In conclusion, our study underlines that an interaction between mononuclear cells and pancreatic stellate cells may contribute to the vicious cycle of inflammation and uncontrolled scarring in chronic pancreatitis.

## Competing interests

The author(s) declare that they have no competing interests.

## Authors' contributions

CWM, AG and ME designed the study, carried out the experiments, analyzed the data and participated in drafting the manuscript. CR and SD participated in the immunohistochemical analyses and the immunoblot experiments. FB participated in the immunohistochemical analyses and histopathological classification of the tissues. TG carried out the quantitative RT-PCR and participated in analyzing the data. BS, HF and NAG conceived of the study, participated in its design and coordination. JK conceived of the study, participated in its coordination and helped to draft the manuscript. All authors read and approved the final manuscript.
